# Cryptogamous Origin of Cholera

**Published:** 1850-01

**Authors:** 


### ARTICLE III.

*Cholera.—Report on the Nature and Import of certain Microscopic Bodies found in the Intestinal Discharges of Cholera.*—Presented to the Cholera Committee of the Royal College of Physicians of London, by their Sub-Committee, on Oct. 17th, 1849.

We propose, in this Report, to lay before the Committee the result of some experimental inquiries on a subject which, within the last few weeks, has engaged much of the attention of the profession. We allude to the discovery, by Mr. Brittan and Mr. Swayne, of Bristol, of peculiar bodies in the "rice-water" dejections of cholera patients; and to the statement that similar bodies have been found by Mr. Brittan in the atmosphere, and, subsequently, by Dr. W. Budd, in the drinking-water of infected localities.

These observations, on account of their important bearing, if true, on the pathology of cholera, seemed to us to demand a searching examination. We have, accordingly, given much time and attention to the subject. Having, in the first place, satisfied ourselves of the distinctive characters of the bodies found in the rice-water dejections, we next sought to verify the observations of Mr. Brittan and Dr. Budd with reference to their presence in the air and drinking-water of places infected with cholera. It was necessary that this part of the inquiry should not be delayed, for the epidemic has already reached its turning point, and it would before long, have been difficult to obtain favorable opportunities for experiments of a satisfactory character.

Our inquiries were afterwards directed to the nature and properties of the newly discovered corpuscles, and to the question of their occurrence in other diseases. In this investigation, we soon perceived that objects totally different had been regarded as identical; but we had arrived at no positive conclusion respecting those which seemed most characteristic of the cholera evacuations, when we received two important communications on the subject from Mr. Marshal and Dr.

Jenner. The letters of these gentlemen are appended to this report; but the results obtained by them are imbodied in it.

Our observation on the air and drinking-water of infected localities, twenty-four in number, gave uniformly negative results. With regard to the value of our experiments, taken separately, it will, we think, appear that many are liable to no objection. Some of those which relate to the drinking-water of infected places, are certainly wanting in the conditions which would make them convincing. But when it is considered that Dr. Budd believes he has detected the objects sought for "in great numbers," in such large bodies of water as the Float, at Bristol, and the Surrey Canal, and that he represents them as being deposited in the sediment of the water, we shall not be thought unreasonable in having expected that they might be discovered in the cisterns of houses and public institutions in which cholera had prevailed severely, although it had ceased for some days or weeks.

Nevertheless, a much larger amount of evidence would have been required to disprove the statements to which our observations refer, had those statements been unassailable from other points. But the facts to be detailed in the subsequent part of this report, will show that the bodies found in rice-water dejections have no peculiar relation to cholera; and, that if they should occasionally be present in the atmosphere, or impure water, this will not happen exclusively, or even especially, in districts infected with the epidemic.

We shall now submit the particulars of all the observations to the Committee, describing, first, those on the air.

[Here follow accounts of the collection of condensed moisture from the atmosphere of apartments where patients had died, or were lying ill with the disease; and of water used by cholera patients in infected localities. These collections were made in twenty-four different neighborhoods where the disease was prevalent.---The following were the results of careful microscopic observation.]

*Observations on Water Condensed from the Atmosphere of Infected Localities.*

Two methods were employed for condensing the aqueous vapor. One was, to suspend in the air to be examined a glass funnel, nearly filled with a freezing mixture, its lower opening having previously been closed with a cork and covered with sealing-wax. The moisture condensed on the out side of the funnel, trickled into a small phial placed beneath.—The second method was, to force air slowly, by means of bellows, through a bent glass tube immersed in ice and salt; when the moisture was deposited on the interior of the tube, and collected in a bulb at its lower part. In either way from half a drachm to a drachm of water was readily obtained.

The water condensed from the air in the several localities, and under the circumstances we have described, was in each case, examined by us more than once. But the search for "annular" bodies, such as those found in the cholera dejections, failed, as we have already intimated. Neither cells, nor rings, nor any thing bearing any resemblance to them could, in most cases, be discovered. We saw merely portions of gelatiniform matter containing bright points; sometimes finely granular, brownish masses, perhaps derived from smoke ---and occasionally colorless, transparent paiticles, of a crystalline appearance, which may have been portions of siliceous dust. After the water had been kept some time, chains of delicate oval vesicles, like those of the torula of yeast, but much smaller, appeared in it. These were absent at first, and could not be mistaken for the cholera discs. Equally unlike those discs were the three or four sepaiate oval cells, which, in two instances, were seen in the water when first examined. They had a clear, single outline, and were not flatteend.

*Microscopic Observations on the Drinking-Water of Infected Places.*

* * * * The uniform result of these experiments, (on the water of infected places,) as of the first series, was negative. No bodies were found which could be regarded as identical with the more characteristic of those found, by Messrs. Brittan and Swayne, in the rice-water dejections of cholera. The objects met with were far more numerous than those seen in the moisture condensed from the atmosphere. The sediment, when viewed with the one-eighth-inch object glass of Ross, or one-sixteenth-inch object-glass of Powell and Lealand, presented, besides amorphous matter, an almost endless variety of organic forms, both animal and vegetable. Amongst these were many round or oval cells, of various dimensions, and some separate rings of minute size, colorless, and pellucid.— The cells had generally very delicate walls and a clear cavity, were never flattened, and often contained a multitude of distinct granules, which, in some instances, presented the molecular motion. Like the rings, these were obviously different in their nature, from the thick-edged discs, which the descriptions and drawings of Messrs. Brittan and Swayne, and Dr. Budd had led us to regard as the characteristic corpuscles of the cholera evacuations.

The negative results of our search in the atmosphere of infected places, for objects identical with those referred to, are confirmed by some observations communicated to us by Mr. Marshall. While cholera was prevalent in St. Giles', he examined the dirt washed from the broken glass of windows, and from cobwebs taken from houses in that district, in which deaths had occurred from four to ten days previously. With one-twelfth-inch or one-eighth-inch object-glass, he found a vast number of objects, such as particles of silex and soot, hairs, wings, and legs of insects, round and oblong cells of a brownish color, very dark spherical granular masses, probably of a confervoid nature, and fragments of vegetable tissue, amongst which were pieces of spiral tubes, and entire rings, apparently of woody tissue, of an oval, polygonal, or circular form. But he detected no discs with double outline. A microscopic examination of the objects collected on a moist surface from the atmosphere of sewers, gave Mr. Marshall a similar negative result with regard to those discoid bodies; although he found (besides fine particles of silex and other dust) brown, oval, and round cells, single and in couplets, minute colorless vesicles, either single, double, or in triplets, a single large oval cell, and numerous opaque, granular, confervoid bodies, of a brownish or blackish green color.

### *Microscopic Observations on the Bodies found in the Cholera Dejections.*

We next proceed to show how various are the bodies which have been confounded together under the terms annular bodies (Mr. Brittan,) cholera cells (Mr. Swayne,) and cholera fungi (Dr. Budd.)

On examining the drawings given by the three gentlemen who have called attention to the subject, four principal forms, which can hardly belong to the same objects, may readily be distinguished.

1. *Rings*, which enclose a free area, and which often are broken. These are usually of minute size, according to Mr. Brittan and Mr. Swayne, but occasionally large according to Dr. Budd.

2. *Globular, or oval cells* chiefly of the middle size, which have a thick wall, with numerous small eminences on its surface and contain a granular mass, in some instances, separated by a clear space from the wall of the cells. These are distinctly figured only by Mr. Swayne, but are regarded by him as perfectly developed cholera cells.

3. Bodies having apparently the form of *discs, with thick rounded edges*, and centres of indistinct structure. These vary extremely in size, including some of nearly the smallest, as well as many of the largest, of the objects represented by the three different observers. They predominate in all the representations given of the corpuscles of the rice-water dejections, and must be taken as the type of the bodies discovered by Messrs. Brittan and Swayne.

4. Large *broken cells*, having apparently homogeneous membranous walls, and containing small, well-defined, oval bodies; figured by Dr. Budd as cholera fungi undergoing decay, but differing in character from all the other objects represented.

A mere inspection of these different figures would suggest strong doubts as to their representing different appearances of really identical bodies in different states or stages of development or decay. The more particular description we have now to give of each kind of body, will demonstrate that they are of various and distinct nature.

1. The rings, when closely examined, are seen to be of different kinds; some perfectly continuous in their entire circle; others formed by a curled fibre; some round, some oval, others lozenge shaped.

Some of these have been traced to their true source by Mr. Marshall, who has found that exactly similar objects may be prepared by the artificial digestion of the vegetables used as food—such as cabbage, potatoes, and onions, the withered style of wheat grain, and portions of cane in sugar ; the spiral and annular tissues of which break down into rings of different sizes or coils resembling rings.

Intermediate between these and the third class of bodies are minute, oval or round, colorless corpuscles, which have an annular appearance ; but, on close inspection, are seen to have their area fielld up with a transparent substance, presenting sometimes perforations. In some specimens of the rice-water fluid, oval bodies, in part having their middle filled up as here described, and, in part, mere rings, exist in extraordinary abundance. The rings of these bodies have been observed, by Mr. Busk and Dr. Griffith, to be divided by cross-lines, into segments, which Mr. Busk thinks are bead-shaped---an appearance which had occasionally been noticed by ourselves as well as by Mr. Marshall. They are calcareous structures, originally derived from chalk, in which they abound; and they have been introduced into the contents of the intestines with the medicines (chalk mixture, aromatic confection, &c.) which the patients have taken.* These minute bodies from the chalk are, of course, not found in all cases ; and we think it not unlikely that, in their absence, the separate nuclei of animal and vegetable structures, as well as the vegetable rings above described, may sometimes have been mistaken for fungi.

2. The globular bodies have been clearly identified by Mr. Marshall with spores of different kinds of uredo, the rust, smut, and bunt of grain; some species of which may be found, not only about the withered style on grains of wheat, but also in almost every specimen of corn and bread.

Mr. Busk has made the same observation, and identifies them with uredo segetum, or bunt.

*It is right to state how we arrived at the knowledge of these facts. Dr. Griffith had pointed out to us that the bodies in question are heavy, polarize light, and are soluble in dilute nitric acid. He suspected that they were oxalate or Phosphate of lime. Mr. Marshall subsequently showed us that acetic acid also dissolves them readily, and that sulphuric acid acts on then, producing needles of sulphate of lime. Having ourselves found the same bodies in the evacuations of two patients suffering from typhoid fever, we were examining them in company with Dr. Griffith and Mr. Marshall, when the demonstration of their calcareous nature reminded us of the fact, that these patients had been taking medicine containing chalk, and at the same, brought to our recollection the remark made to one of us by Mr. Topping, that Mr. Brittan's "annular bodies" were to be found in chalk-mixture. Accordingly, we examined a portion of medicine containing aromatic confection, and, afterwards, a piece of common chalk, and in both, found the bodies described above, though not the larger discs which are also found in the rice-water fluid. Ehrenberg figures these calcareous bodies, and describes them as being "crystalloids." Abh. d. Akad. d. Wiss. z. Berl. 1838, p. 68.

3. Discs, with thick, elevated, and somewhat irregularly curved margins; the central area flattened, and obscurely granular. They have generally a yellowish, or pale brown tint, which varies in depth with the color of the fluid containing them. These are the most peculiar of the bodies found in cholera, and differ from the rest in being more or less soluble in ether. Mr. Marshall, who first informed us of this fact, found that the smaller discs undergo nearly complete solution, leaving a cavity in the dried mucus, whilst the larger ones leave a fine granular film. They are apt to break across, and the thick margin to curl inwards. They are evidently not cells, nor have they any organized structure which could give them any claim to be regarded as living organisms. On the other hand, their solubility in ether shows that they consist, in great part, of some substance of the class to which the fats, resins, and saponaceous matters belong. This observation led Mr. Marshall to examine different fatty snbstances, and at length to find that curled concretions, not unlike the discs found in cholera, could be obtained by compressing a piece of rich cheese (with or without the addition of ether) between two plates of glass. We are not yet able to account for the origin of these peculiar discs. Mr. Busk regards the smaller ones as altered starch grains. It is, at all events, certain that they are not fungi; and, as we shall afterwards see, that they are not peculiar to cholera.

Mr. Busk thinks that the larger discs are the altered contents of bran-cells. Mr. Marshall, too, has, independently, made the observation, that certain yellowish bodies, sometimes seen, which have a thinner and narrower border than the fatty discs, and are merely rendered pellucid by ether, may, perhaps, be derived from bran. The granular masses contained in bran-cells have, however, when undigested, no distinct border.

4. Under the fourth class of bodies, we refer to those represented by Dr. Budd as the cholera fungi, undergoing decay and disintegration. They are evidently of of a different nature from those figured by him as characteristic of the fresh cholera dejections. The mode of disintrgration of the two classes of bodies is quite distinct: the so-called cholera bodies, after resisting the action of water for some time, break up into irregular granular masses; whilst the decomposing bodies depicted by Dr. Budd, seem to be, in part, homogeneous, membranous cells dehiscing; and are, perhaps, starch cells. The rings are most probably, parts of disintegrated vegetable tissue.

It is shown by Mr. Marshall, and had before been noticed by Boehm, and others, that cells like fungi, or their spores, are occasionally found in the excretions in cholera.—These, however, have a more delicate structure than any of the bodies described as characteristic of cholera, and are totally different from them. It is well known that various vegetable forms are apt to become developed in organic fluids generally.

From a review of the foregoing facts, it is obvious that various bodies found in cholera dejections have been confounded and described as identical. It is also shown, that many are traceable to an extraneous source, and that even the discs placed in our third division, are not fungi. The statement, that the bodies found in the cholera dejections present an endogenous multiplication, has, in all probability, arisen from confounding them with the uredo, or from mistaking the appearances produced by the small bodies seen through, or upon, the larger ones, or entangled in their substance.

We are unable to identify the rings obtained from the air, and figured by Mr. Brittan, with any of the bodies included him under the term "annular bodies." Our own experiments have satisfied us that these bodies do not commonly exist in the atmosphere of infected places, but the observations of Mr. Marshall, on the dirt collected from windows and cobwebs, show the great variety of matters which must be wafted about in the air, in the form of dust, and which might, in different instances, be caught with the condensed moisture.

The bodies represented by Dr. Budd, as being found in impure drinking water, have the form of discs with thick edges. We have ourselves never seen such bodies in water. But, if it should be established that the contents of bran-cells sometimes assume that form, the occasional presence, in water, of bodies capable of being confonnded with the discs derived from the discharges of cholera, will not appear remarkable.

Had the bodies described by Messrs. Brittan and Swayne been proved proved by the foregoing investigations, to be of fungoid nature, yet the facts we have now to add would have shown that they have no necessary connection with cholera. In the first place, they seem not to be constantly present in the discharges. It is, indeed, remarkable that, in those dejections which, from the absence of color, have usually been regarded as the characteristic of the disease, they are frequently absent. We have failed to find them in several instances. In one, a portion of every evacuation was set apart, and examined several times by each of us, and yet in no portion could we detect them.

A still more important fact, which, from the explanations already given might be anticipated, is that all the more remarkable of the bodies which have been thought peculiar to cholera, exist in the intestinal evacuations of persons affected with other diseases. Dr. Jenner first demonstrated to us their presence, in great abundance, in the dejections of a patient affected with typhoid fever. We have since verified his observations in five other cases of this disease. We have also satisfied ourselves of the existence of some of the forms in dejections apparently healthy, from two patients in Guy's Hospital; one suffering from bronchitis, the other from early cirrhosis of the liver; and Mr. Marshall has detected small annular bodies, "in the mucus covering the healthy excrement" of several herbiverous animals. It is obvious that bodies derived from such various sources will not commonly be found all present together. This, indeed, is not the case in cholera. The minute bodies, especially, which belong to chalk will, of course, very rarely be met with, except that substance has been taken as medicine.

We shall now briefly re-state the principal results we have arrived at, and submit the conclusion which seems to us justified by them.

1. Bodies presenting the characteristic forms of the so-called cholera fungi are not to be detected in the air, and as far as our experiments have gone, not in the drinking water of infected places.

2. It is established that, under the terms "annular bodies," "cholera cells," or "cholera fungi," there have been confounded many objects of various, and totally distinct natures.

3. A large number of these have been traced to substances taken as food or medicine.

4. The origin of others is still doubtful, but these are clearly not fungi.

5. All the more remarkabl- forms are to be detected in the intestinal evacuations of persons laboring under diseases totally different in their nature from cholera.

Lastly, we draw from these premises the general conclusion, that the bodies found and described by Messrs. Brittan and Swayne are not the cause of cholera, and have no exclusive connection with that disease;—in other words, that the whole theory of the disease which has recently been propounded, is erroneous, as far as it is based on the existence of the bodies in question. [Signed]

William Baly, M.D.,
William W. Gull, M.D.,
*Cholera Sub-Committee.*

---

